# The effectiveness of early colchicine administration in patients over 60 years old with high risk of developing severe pulmonary complications associated with coronavirus pneumonia SARS-CoV-2 (COVID-19): study protocol for an investigator-driven randomized controlled clinical trial in primary health care—COLCHICOVID study

**DOI:** 10.1186/s13063-021-05544-7

**Published:** 2021-09-06

**Authors:** Elena Bustamante Estebanez, Lucía Lavín Alconero, Beatriz Josa Fernández, Monica Gozalo Marguello, Juan Carlos López Caro, Jonathan Diez Vallejo, Marta Fernandez Sampedro, Pedro Muñoz Cacho, Carlos Richard Espiga, María Mar García Saiz

**Affiliations:** 1 Management of primary health care centers, Area I, Area II, Area III and Area IV, Servicio Cantabro de Salud, C. Vargas 57, 39010 Santander, Cantabria Spain; 2 Marqués de Valdecilla Research Institute (IDIVAL), s/n, Calle Cardenal Herrera Oria, 39012 Santander, Cantabria Spain; 3 Clinical Trials Agency Valdecilla-IDIVAL, Marqués de Valdecilla University Hospital, Av. Valdecilla, 25, 39008 Santander, Cantabria Spain; 4 Department of Microbiology, Marqués de Valdecilla University Hospital, Av. Valdecilla, 25, 39008 Santander, Cantabria Spain; 5 Department of Infectious Diseases, Marqués de Valdecilla University Hospital, Av. Valdecilla, 25, 39008 Santander, Cantabria Spain; 6 Department of Community Health, Servicio Cantabro de Salud, C. Luis Vicente de Velasco 1, 39011 Santander, Cantabria Spain; 7 Emeritus Doctor, Department of Hematology, Marqués de Valdecilla University Hospital, Av. Valdecilla, 25, 39008 Santander, Cantabria Spain; 8 Department of Clinical Pharmacology, Marqués de Valdecilla University Hospital, Av. Valdecilla, 25, 39008 Santander, Cantabria Spain

**Keywords:** Coronavirus, Colchicine, Primary health care, Early treatment, No hospitalized

## Abstract

**Background:**

There is no strong evidence that any drug is beneficial either for the treatment of SARS-CoV-2 disease or for post-exposure prophylaxis. Therefore, clinical research is crucial to generate results and evaluate strategies against COVID-19. Primary care (PC) centers, the first level of care in the health system, are in a favorable position to carry out clinical trials (CD), as they work with a large volume of patients with varied profiles (from acute to chronic pathologies). During the COVID-19 pandemic, the need for hospital admission and mortality is higher in people > 60 years. Therefore, this is a target population to try to reduce the serious complications and lethality of COVID pneumonia and to avoid overloading the hospital system. Given the pharmacological properties of colchicine (anti-inflammatory and anti-fibrotic, possible inhibition of viral replication, and inhibitory effect on coagulation activation), early treatment with colchicine may reduce the rate of death and serious pulmonary complications from COVID-19 in vulnerable patients.

**Methods:**

The COLCHICOVID study is a randomized, multicenter, controlled, open-label parallel group (2:1 ratio), phase III clinical trial to investigate the efficacy of early administration of colchicine in reducing the development of severe pulmonary complications associated with COVID-19 infection in patients over 60 years of age with at-risk comorbidities.

**Discussion:**

This is a pragmatic clinical trial, adapted to usual clinical practice.

The demonstration that early administration of colchicine has clinical effectiveness in reducing the complications of SARS-CoV-2 infection in a population highly susceptible may mitigate the health crisis and prevent the collapse of the health system in the successive waves of the coronavirus pandemic. In addition, colchicine is a well-known medicine, simple to use in the primary care setting and with a low cost for the health system.

**Trial registration:**

ClinicalTrials.govNCT04416334. Registered on 4 June 2020. Protocol version: v 3.0, dated 22 September 2020.

## Administrative information

**Table:** 

Title {1}	THE EFFECTIVENESS OF EARLY COLCHICINE ADMINISTRATION IN PATIENTS OVER 60 YEARS OLD WITH HIGH RISK OF DEVELOPING SEVERE PULMONARY COMPLICATIONS ASSOCIATED WITH CORONAVIRUS PNEUMONIA SARS-CoV2 (COVID19): Study protocol for an investigator-driven randomised controlled clinical trial in PRIMARY HEALTH CARE. COLCHICOVID-STUDY
Trial registration {2a and 2b}.	Clinical trials NCT04416334, Registered on 4 June 2020, https://clinicaltrials.gov/ct2/show/NCT04416334
Protocol version {3}	Version 3.0 del 22^th^ de September 2020
Funding {4}	This study is a non-commercial study. A local grant called PRIMARY CARE RESEARCH PROJECTS SUPPORT PROGRAM (PRIM-VAL) 2020 has been received and published in the autonomic Call for Programs to Revitalize Biosanitary Research in 2020 and the call is specifically set up for COVID-19 related projects as BOC 24 March 31st 2020 Resolution states.
Author details {5a}	^1^Marqués de Valdecilla Research Institute (IDIVAL), s/n, Calle Cardenal Herrera Oria, 39012 Santander, Cantabria, Spain ^2^Department of Clinical pharmacology, Marqués de Valdecilla University Hospital, Av. Valdecilla, 25, 39008 Santander, Cantabria, Spain ^3^Clinical trials Agency Valdecilla-IDIVAL, Marqués de Valdecilla University Hospital, Av. Valdecilla, 25, 39008 Santander, Cantabria, Spain ^4^Management of primary health care centers, Area I, Area II, Area III and Area IV. Servicio Cantabro de Salud. C. Vargas 57, 39010 Santander, Cantabria, Spain. ^5^Department of Microbiology, Marqués de Valdecilla University Hospital, Av. Valdecilla, 25, 39008 Santander, Cantabria, Spain. ^6^Department of Infectious diseases, Marqués de Valdecilla University Hospital, Av. Valdecilla, 25, 39008 Santander, Cantabria, Spain ^7^Department of Community Health, Servicio Cantabro de salud. C. Luis Vicente de Velasco 1, 39011 Santander, Cantabria ^8^Emeritus Doctor, Department of Hematology, Marqués de Valdecilla University Hospital, Av. Valdecilla, 25, 39008 Santander, Cantabria, Spain
Name and contact information for the trial sponsor {5b}	IDIVAL (Instituto de investigación sanitaria Marques de Valdecilla) Dr. Francisco Galo Peralta as representative of the sponsor direccion@idival.org
Role of sponsor {5c}	The authors declare that they have no competing interests. The study was designed and conceived independently from the funding and sponsor institutions, neither of which had a role in the collection, management, analysis or interpretation of data or the writing of the final manuscript.

## Introduction

### Background and rationale {6a}

Since the beginning of the SARS-CoV-2 coronavirus pandemic in Spain, there has been a high incidence in the older population, with a tendency to develop serious complications and greater lethality [1, 2]. In addition, this population group presents higher rates of admission, estimated at 15–20% for patients between 60 and 70 years of age and increasing to 20–30% in those over 70 years of age [3]. Currently, 60% of those hospitalized are over 70 years old, and it is estimated that 15–20% will need to be admitted to the ICU and that the mortality rate will be 14–22%.

In Cantabria, with an estimated population of 570,000 inhabitants, there are about 90,000 people over 70 years old. Of the 90,000 people, it is estimated that about 20–40% will be infected and half of them may develop severe pneumonia requiring hospitalization and ICU admission for respiratory failure. Therefore, this is a target population with a need to minimize or avoid the severe complications of COVID pneumonia and its lethality and minimize, as much as possible, an overload of the hospital health system.

Colchicine is a drug of the tricyclic alkaloid family extracted from *Colchicum* plants (*Colchicum autumnale*) [4]. This drug has powerful anti-inflammatory effects, which has made it the treatment of choice for various rheumatological diseases (gout, familial Mediterranean fever, and Behcet’s disease). In addition, it has been used in cardiovascular pathologies (pericarditis, acute coronary syndrome, auricular fibrillation) due to its anti-inflammatory and anti-fibrotic properties [5, 6]. The mechanisms through which colchicine exercises its anti-inflammatory properties are multiple. Perhaps the most relevant of these mechanisms is the ability of colchicine to bind to free tubulin dimers in the cells with the formation of a tubulin–colchicine complex [7, 8], and the subsequent inhibition of microtubule polymerization [9].

Other effects of colchicine not so clearly related to the depolymerization of microtubules are the inhibition of adhesion, extravasation, and recruitment of neutrophils by altering the expression of L-selectin in neutrophils and the distribution of E-selectin in endothelial cells [10]. Finally, and based on studies carried out in microcrystalline arthropathies, it has been shown that colchicine suppresses the activation of the inflammasome (NLRP3) induced by calcium pyrophosphate and monosodium urate crystals. This suppression inhibits the activation of caspase-1 and subsequent release of IL-1b and IL-18 [11].

On the other hand, in addition to its marked anti-inflammatory and anti-fibrotic properties [12], colchicine has shown, at least in vitro, its effectiveness as an inhibitor of viral replication [13–15]. Treatment with colchicine has shown clinical efficacy in a small series of patients with severe myocarditis [16] and pericarditis of viral etiology [17]. In addition, viroporins are viral proteins that alter the permeability of the membrane and can trigger the activation of the inflammatory cascade [18]. These viral proteins may be involved in different stages of the virus infection cycle, including cell entry, replication, morphogenesis, and release from the infected cell. Although information is scarce, previous studies have shown that viroporin E [19], 3a [20], and 8a [21], components of the SARS-associated coronavirus (SARS-CoV), are responsible for forming calcium-permeable ion channels and activating the NLRP3 inflammasome. Thus, at least theoretically, the blockade of the inflammasome with colchicine could diminish the inflammatory process triggered by the viral infection.

Like other members of the Coronaviridae family (MERS-CoV, SARS-CoV), SARS-CoV-2 is able to bind to the angiotensin II enzyme receptor [22] and to enter the cell by different mechanisms including endocytosis [23]. After transcription and replication of the viral genome, translation of viral protein, and viral assembly, the multiple copies of coronaviruses produced are released to the exterior through a mechanism of exocytosis which, like endocytosis, is a mechanism dependent on intracellular microtubules [24, 25]. As in other infectious diseases, viral antigens are recognized by antigen-presenting cells capable of triggering the inflammatory response. Although the mechanism of antigen recognition and presentation is not well defined in SARS-CoV-2 infection, it is likely, as with other viruses, to be Toll-like receptor (TLR) dependent [26]. TLRs in turn require intracellular microtubules [27] and are related to the inflammatory response in other infections [28]. Overall, it is observed that microtubules are involved in the different stages of SARS-CoV-2 infection at the cellular level, and therefore, colchicine could have an important regulatory effect.

Clinically, SARS-CoV-2 causes COVID-19 disease, which can occur in two phases. The first phase is related to the infection and viral replication and can go unnoticed or manifest as a banal or paucisymptomatic viriasis. Approximately 15% of patients will develop a second phase, characterized by the development of pneumonia that can evolve into an acute respiratory distress syndrome and potentially fatal multiorgan failure [29]. This second phase has been directly linked to a massive inflammatory response characterized by an increase in pro-inflammatory cytokines (IL-1, IL2, IL-6, IL-8, IFN-, etc.) [30, 31] which produce a hyperstimulation of the mononuclear–monocytic system, visible at the level of laboratory tests by an increase in the acute phase reactants such as PCR and ferritin. Secondarily, the release of cytokines conditions the injury of the capillary alveolar membrane giving rise to respiratory distress [30] and favors a procoagulant state and hyperfibrinolysis characterized by an increase in D-dimer [32, 33]. In this sense, colchicine seems to have an inhibitory effect on coagulation activation through its effect on platelets [34, 35] which could be beneficial in this disease.

On the other hand, in addition to the respiratory component, a clear risk for the cardiovascular system has been described in patients with severe SARS-CoV-2 infection. Recently, it has been reported that a significant proportion of patients with severe SARS-CoV-2 infection may have myocardial and endocardial involvement, with cardiac involvement being key to the prognosis of these patients [36–38]. In this sense, colchicine has also demonstrated its beneficial effect in various syndromes of cardiovascular origin, mainly through the reduction of its inflammatory component [39, 40]. It is important to highlight that, in a recent trial [41] with low doses of colchicine administered to patients with a history of acute coronary syndrome, a statistically significant reduction in cardiovascular complications was demonstrated.

Recently, after the design of our clinical trial, the efficacy of colchicine administration was analyzed in several studies. To this day, only three clinical trials have been published, two in patients admitted to hospital with COVID-19 and the last one in an outpatient setting. After a randomized, double-blinded, placebo-controlled clinical trial of colchicine for the treatment of moderate to severe COVID-19, Lopes et al. concluded that treatment with colchicine for 5 days reduced the length of both, supplemental oxygen therapy and hospitalization [42]. In the study GRECCO-19, an open-label, randomized clinical trial, participants who received colchicine administration, for as long as 3 weeks, had statistically significantly improved time to clinical deterioration [43]. Only one randomized, double-blinded, adaptive, placebo-controlled, multicenter trial (COLCORONA) showed that colchicine treatment for 30 days can be used in community-treated patients to prevent hospitalization, reducing the severity and mortality caused by the disease [44]. Other epidemiological studies have shown the beneficial effects of colchicine treatment in COVID-19 when given early in the course of the disease [45–50].

However, a systematic review involving 5778 patients concluded that further randomized clinical trial studies are still needed to confirm the beneficial results [51]. In our view, patients with a mild-moderate clinical presentation may not initially require hospitalization, and most patients will be able to manage their illness at home, but early anti-inflammatory treatment, such as colchicine, could prevent the progression to severe illness.

Therefore, a pragmatic randomized clinical trial is proposed to demonstrate that colchicine is effective and safe as an early treatment for reducing the clinical complications associated with coronavirus disease.

## Objectives {7}

### Hypotheses

Given the pharmacological properties of colchicine (anti-inflammatory and anti-fibrotic, possible inhibition of viral replication, and inhibitory effect on coagulation activation), early treatment with colchicine may reduce the rate of death and serious pulmonary complications from COVID-19 in vulnerable patients over 60 years of age affected by COVID-19 and with comorbidities at risk.

### Objectives

- To determine if early treatment with colchicine reduces the rate of death and serious pulmonary complications related to COVID-19 in patients over 60 years of age with at-risk comorbidities measured as the number of participants who died or are hospitalized in the 30 days after randomization
- To determine the safety of colchicine treatment in this patient population

## Trial design {8}

The COLCHICOVID study is a randomized, multicenter, controlled, open-label parallel group (2:1 ratio), phase III clinical trial to investigate the efficacy of early administration of colchicine in reducing the development of severe pulmonary complications associated with COVID-19 infection in patients over 60 years of age with at-risk comorbidities.

This is a pragmatic clinical trial, adapted to usual clinical practice.

## Methods: participants, interventions, and outcomes

### Study setting {9}

Cantabria, a region in the north of Spain, is territorially structured in four primary care areas, according to the Decree 66/2001 of August 17, establishing the Autonomous Health Map of Cantabria: Area I (Santander), Area II (Laredo), Area III (Reinosa), and Area IV (Torrelavega). The clinical trial is carried out in 42 health centers in Cantabria, with each health area is represented by a principal researcher.

The PCR tests will be carried out in the Marqués de Valdecilla Hospital where the principal researcher and the clinical study coordinator are located.

The Clinical Trials Agency of IDIVAL (Valdecilla Biomedical Research Institute) and SCReN (Spanish Clinical Research Network) were responsible for obtaining authorization from the local Ethics Committee (EC) and the Spanish Agency of Medicines and Medical Devices (AEMPS) of the start-up of the study.

### Eligibility criteria {10}

Patients will be detected through the Microbiology Service Database from Hospital Universitario Marqués de Valdecilla, where all local PCR determinations for SARS-CoV-2 are centralized.

It will be essential to have a diagnosis of COVID-19 infection confirmed by PCR and that patients start treatment in the first 72h with or without symptoms. Candidates will not be assessed with:

- Previous positive PCR
- Routine positive PCR by scheduled operation or procedure or by chance

All patients who meet all the criteria for inclusion and none for exclusion will be asked for informed consent. In case of obtaining it, we will proceed to the randomization. The randomization will be centralized and will be available either by telephone or online using the web platform Research Electronic Data Capture (REDCap). It will be done in a 2:1 allocation ratio to receive colchicine and symptomatic treatment, or symptomatic treatment for 21 days.

Patients must meet all of the following criteria in order to participate in the study.

### Inclusion criteria

1. Patients of both sexes who are at least 60 years old
2. Diagnosis of COVID-19 infection in the last 72 h, confirmed by PCR
3. Outpatient follow-up (not hospitalized or under consideration) or institutionalized in nursing homes/residences
4. At least two of the following high-risk criteria:
  - 60 years of age or older
    and
  - Any of the following: diabetes mellitus, high blood pressure, known pulmonary disease (including asthma or chronic obstructive pulmonary disease), known heart failure, known coronary disease, bicytopenia, pancytopenia, or the existence of simultaneous neutrophilia and lymphopenia
5. Patients must be able and willing to comply with the requirements of this study protocol

### Exclusion criteria

1. Hospitalized patients or under immediate consideration of hospitalization
2. Patient taking colchicine for other indications
3. History of allergic reaction or sensitivity to colchicine
4. Inflammatory bowel disease, gastric ulcer, chronic diarrhea, or malabsorption
5. Pre-existing progressive neuromuscular disease
6. Kidney damage and estimated glomerular filtration rate < 30 ml/min/1.73m^2^
7. Undergoing chemotherapy for cancer, including hematological malignancies
8. Treatment with CYP3A4 and/or glycoprotein inhibitor drugs
9. Immunosuppressive treatment
10. History of cirrhosis, chronic active hepatitis, severe chronic disease defined by AST or ALT values exceeding 3 times the upper limit of normal
11. If the investigator considers to be an inadequate candidate, for any reason

The criteria for withdrawal or dropout criteria are described in item 11b of the present manuscript.

### Who will take informed consent? {26a}

Principal investigators in each area provide informed consent to patient candidates for participation. The latest version approved of the patient’s information sheet will be signed by all selected subjects before inclusion in the clinical trial.

In the case of selected subjects who cannot understand the study or do not have the ability to read or write, the informed consent must be provided by the legal representative or use verbal consent before any procedure of the study is carried out.

### Additional consent provisions for collection and use of participant data and biological specimens {26b}

The biological samples of serum or excess DNA will be kept in the IDIVAL biobank. The patient must sign the informed consent sheet to transfer the samples to the study.

In accordance with current legislation RD 1716/2011, on basic requirements for the authorization and operation of biobanks for biomedical research purposes and the treatment of biological samples of human origin, and regulating the operation and organization of the National Register of Biobanks for biomedical research, Chapter I of Title II Storage and conservation of biological samples for human use, the samples will be conserved for the duration of the research and will therefore be destroyed after the closing visit, data analysis, and publication of results.

## Interventions

### Explanation for the choice of comparators {6b}

Most pre-symptomatic or mildly ill patients can be managed in an ambulatory setting. WHO recommends that patients with COVID-19 receive treatment for their symptoms, such as antipyretics for fever and pain as well as adequate nutrition and appropriate rehydration [52]. The selection of symptomatic treatment as the comparator or control group is therefore justified.

### Intervention description {11a}

The intervention and comparator description is detailed in Table 1.

**Table 1:** Intervention description

Groups	Interventions
**Experimental group**	*Colchicine plus symptomatic treatment (paracetamol)* Patients in this arm will receive study medication colchicine 0.5 mg orally (PO) twice, daily for the first 3 days and then once daily for the last 18 days. If a dose is missed, it should not be replaced. All patients should also receive the best symptomatic treatment (mainly paracetamol), based on clinical practice.
**Control group**	*Symptomatic treatment* Symptomatic treatment (paracetamol or best symptomatic treatment) based on doctor recommendations.

### Criteria for discontinuing or modifying allocated interventions {11b}

In accordance with the current revision of the Declaration of Helsinki (Fortaleza, Brazil October 2013), and with applicable regulations, a patient has the right to withdraw from the study at any time and without giving any reason, without compromising their current and future clinical care.

A patient may be withdrawn from the study in the following cases:

- ◦ The patient withdraws the consent
- ◦ For safety reasons: if adverse events appear that, due to their type or seriousness, make it necessary for the patient not to remain in the study
- ◦ Deterioration of the general condition, new-onset respiratory failure (dyspnea), in which case they will be admitted to the hospital
- ◦ Toxicity/intolerance to colchicine
- ◦ Loss in follow-up
- ◦ The patient is not willing to comply with the procedures required in the protocol

In any case, the causes that have led to the dropout or withdrawal of the patient from the study will be recorded in detail in the electronic case report form (eCRF). In both cases, the patient will continue to make the scheduled visits to evaluate the safety and tolerability of the treatment unless the subject expresses a contrary wish. For the rest, once the patient is not in the study, he or she will be attended by the corresponding doctor according to the usual clinical practice.

### Strategies to improve adherence to interventions {11c}

To verify adherence to treatment and safety, the patient will be followed up every 48 h via telephone calls.

### Relevant concomitant care permitted or prohibited during the trial {11d}

The concomitant use of drugs that inhibit CYP3A4 and/or glycoprotein P (GpP) during the follow-up of the trial is contraindicated due to the risk of interaction and increased toxicity of colchicine.

### Provisions for post-trial care {30}

This is a non-commercial study carried out by Spanish researchers, employees of the national public health system. The sponsor of the study is a non-profit organization, IDIVAL Health Research Institute.

There is no compensation for the subjects participating in the clinical trial.

### Outcomes {12}

The primary outcome will be a composite measure of death or the need for hospitalization due to COVID-19 infection, defined as the proportion of participants who die or require hospitalization within 30 days following randomization.

The secondary endpoint measures are the occurrence of death, the need for hospitalization, the need for admission to the critical care unit, and the need for mechanical ventilation in the 30 days after randomization.

Improvement or worsening of clinical and analytical parameters will be assessed by the ordinal WHO scale that measures illness severity over time.

The safety of the treatment will be assessed by recording adverse events/serious adverse events on each of the study visits. Signs and symptoms, as well as laboratory alterations in the patients treated, will be collected.

All patients included in the COLCHICOVID study are provided with indications for self-monitoring:

i. The temperature should be taken and recorded twice a day (in the morning and late afternoon), before taking an antipyretic or analgesic to avoid masking the fever
ii. Monitoring for worsening signs and symptoms or the appearance of new ones
iii. Monitoring for signs and symptoms of toxicity if appropriate
iv. The assessment of the outcome variables, both of efficacy and safety, will be conducted through telephone follow-up every 48 h, and in person on the 10th, the 21st, and then monthly until the end of the patient in the study

If there are warning signs and symptoms: dyspnea at rest or from minimal exertion, pleuritic pain, hemoptysis, altered level of consciousness, diarrhea with dehydration, unconscious vomiting, or high refractory fever, the patient will be evaluated in person, if possible, and a transfer to the hospital will be considered.

If serious side effects or intolerance appears, treatment suspension and patient withdrawal from the clinical trial will be assessed.

### Participant timeline {13}

The flow chart for the study and the schedule of visits and assessments at different points in the study protocol are described in Table 2.

**Table 2:** Schedule of visits and assessments at different points of the study protocol

Assessments	Study period			
	Screening	Visit 1 (day 10) ± 3 days	Visit 2 (day 21) ± 3 days	Visit end (day 61) ± 3 days
		The visits are made by *telephone* call every 48 h and face-to-face visits on the 10th day and 21st day of the treatment and monthly until the end of the patient in the study.		
Informed consent form	x			
Randomization	x			
Medical history	x	x	x	x
Vital signs	x	x	x	x
Physical examination	x			
Diagnostic PCR	x			
Blood analysis	x*	x	x	
Treatment of the clinical trial		x	x	
Concomitant medication, including vaccination schedule	x	x	x	x
Adverse events		x	x	x

x* basal blood analysis valid up to 3 months before selection

### Sample size {14}

According to the literature [3], an estimated incidence of the primary outcome of 10% in the group treated with placebo and 5% in the group treated with colchicine, in a bilateral contrast, for an alpha risk of 5% and a beta risk of 80%, with a proportion of 2/1 between the colchicine/placebo groups, 636 and 318 are required in the groups treated with colchicine and placebo, respectively.

Randomization will be stratified by sex and age groups: 60–70, 70–80, and 81 and more years.

In the current COVID daily cases from the primary health care areas, it is estimated about 5–10 cases of patients to randomize.

The total number of patients to be included is 954 patients.

### Recruitment {15}

In the current circumstances of the COVID-19 pandemic, 10–20 cases of coronavirus-positive PCR patients are being detected daily. Participants will be recruited from the Microbiology Service Database that contains the patients with a positive PCR result elaborated daily with data from all the region. Candidates will not be selected if they have a positive PCR test at an earlier date, or if the result was obtained by a routine pre-surgical determination or by chance.

The initiation of vaccination in the selected age group is not considered an exclusion criterion in the study.

Data will be collected in the CRF on the vaccination schedule administered to the participants.

Weekly meetings with the research team in order to resolve possible limitations and dissemination of the information regarding the clinical trial in local press are strategies to improve recruitment.

## Assignment of interventions: allocation

### Sequence generation {16a}

Participants will be randomly assigned to the experimental or control group with a 2:1 assignment according to a computer-generated randomization schedule stratified by sex and age groups: 60–70, 70–80, and 81 and over. Randomization codes will be assigned strictly sequentially in each stratum as subjects become eligible for randomization. The block sizes will not be disclosed, to ensure concealment.

The randomization list is included in the eCRF and only the administrative staff of the Research Electronic Data Capture (REDCap) base has access to it. Primary care investigators do not have access to the randomization list.

### Concealment mechanism {16b}

The principal investigator and clinical coordinator located at the Marqués de Valdecilla University Hospital carry out the first screening of the list of positive PCR patients provided by the Microbiology Service. The screened list is then sent to primary care investigators to verify the criteria of patients to be included in the clinical trial.

Once the primary care investigator checks that the patient is a candidate for inclusion in the study, a signature of the informed consent form and automatic central randomization will be performed either by telephone or on the online web platform Research Electronic Data Capture (REDCap) using the electronic CRF.

### Implementation {16c}

The code of each patient included in the COLCHICOVID trial will be generated by the eCRF, REDCap, and consists of a first digit relating to the primary care area (Area I: number 8, Area II: number 9, Area III: number 10, Area IV: number 11) and a second digit corresponding to the consecutive number of the patient included.

The assignment of the experimental or control group will be carried out by the randomization module of REDCap, which previously included the randomization list generated by the statistical program STATA (StataCorp. 2017. *Stata Statistical Software: Release 15*. College Station, TX: StataCorp LLC). Allocation is stratified by age groups (60–70, 70–80, and 81) and sex (male/female).

## Assignment of interventions: blinding

### Who will be blinded {17a}

Due to the nature of the intervention, neither trial participants nor primary care researchers can be blinded to allocation; however, one of the investigators will blindly assess in each patient the primary outcome of the study, mainly if the hospital admission criteria are met.

The intermediate and final analyses will be carried out by the study statistician. An independent group composed by the principal investigator, the coordinating investigator, and the statistician will make decisions on the progress of the study based on the results. The medical staff involved in the direct recruitment and follow-up of patients will not be engaged in this task in order to maintain a blinded analysis.

### Procedure for unblinding if needed {17b}

Not applicable

## Data collection and management

### Plans for assessment and collection of outcomes {18a}

Patient-reported clinical status and outcome data will be collected at baseline and throughout the study.

In addition to mortality, the following criteria for hospital admission, outcome of the study, have been previously defined: development of acute respiratory failure, evidence of crackles suggestive of interstitial pneumonia of infectious origin, presence of dyspnea and/or tachypnea > 20 breaths/min, occurrence of serious adverse events related or not with medication under study, axillary temperature greater than or equal to 38°C maintained or persistent for more than 72 h, and clinical signs (cough, dyspnea, fever, asthenia, myalgia, arthralgia, anosmia, hypogeusia, pleuritic pain...) persistent and without clinical improvement after 5–7 days of control by primary care physician.

### Plans to promote participant retention and complete follow-up {18b}

This protocol was developed as a preventive measure to avoid complications caused by the COVID-19 disease in an emergency context due to the pandemic, so the duration of treatment and follow-up are limited to only 21 days and 2 months, respectively. Patient abandonment is not contemplated, but if this happens, it will be followed according to the usual clinical practice.

### Data management {19}

The Research Electronic Data Capture (REDCap) system will be used for data collection and query handling.

Data collection will be done by the doctors involved in the study, and the data obtained will be recorded on the eCRF as specified in the study protocol and in accordance with the instructions provided. The review of the data is carried out by the clinical research associate (CRA) from the Spanish Clinical Research Network (SCReN) according to the monitoring plan to ensure the validity of the results obtained.

The investigators will ensure the accuracy, completeness, and timeliness of the data recorded and of the provision of answers to data queries. The investigator will sign the completed eCRFs. A copy of the completed eCRFs will be archived at the study site upon completion of the study.

### Confidentiality {27}

Personal data will be processed in accordance with Regulation (EU) 2016/679 of the European Parliament and of the Council of 27 April 2016 on the protection of individuals with regard to the processing of personal data and on the free movement of such data, and the relevant local laws.

The data collected for the study shall be identified by an alphanumeric code in such a way that it is not possible to identify the patient. Only the investigator and authorized persons involved in the study will have access to this code and undertake to use this information exclusively for the purposes of the study. All the generated data will be recorded in the CRF in an anonymized form.

Members of the Clinical Research Ethics Committee or Health Authorities may have access to this information in compliance with legal requirements. The confidentiality of this data will be preserved and it cannot be linked to personal data, even if the results of the study are published.

### Plans for collection, laboratory evaluation, and storage of biological specimens for genetic or molecular analysis in this trial/future use {33}

The residual samples after completing the mandatory blood tests on the patients included may be stored in an ad hoc collection in the IDIVAL biobank of biological samples for purposes related to clinical research. Informed consent designed for this purpose will be obtained. If a subject withdraws consent to the use of their donated biological samples, those samples will be destroyed, and the action documented.

## Statistical methods

### Statistical methods for primary and secondary outcomes {20a}

The intervention arm (colchicine and symptomatic treatment) will be compared against the control (symptomatic treatment) for all primary analysis. For the evaluation of the primary and secondary outcomes, the chi-squared test or exact Fisher test will be used, since all the variables involved are categorical (outcomes related to death, hospitalization, and adverse effects); the comparison will be made in independent groups. For all tests, we will use 2-sided *p*-values with alpha < 0.05 level of significance. All safety parameters will be analyzed descriptively.

The SPSS v25 (IBM Corp. Released 2017. BM SPSS Statistics for Windows, version 25.0. Armonk, NY: IBM Corp.) statistical package will be used to conduct analysis.

### Interim analyses {21b}

Intermediate analyses will be carried out monthly for the duration of the study. There will be a follow-up at 1 year, to evaluate negative medium/long-term effects of colchicine.

The independent recruitment committee will decide if it is appropriate to continue the clinical trial.

### Methods for additional analyses (e.g., subgroup analyses) {20b}

Not applicable

### Methods in analysis to handle protocol non-adherence and any statistical methods to handle missing data {20c}

To prevent attrition bias, outcome data obtained from all participants will be included in the intention-to-treat analysis, considering all patients as randomized regardless of whether they received the randomized treatment. Secondarily, an analysis per protocol will be carried out with the patients who have complied with the protocol.

### Plans to give access to the full protocol, participant-level data, and statistical code {31c}

Dissemination of results directed to patients will be channeled through the Spanish Agency for Medicines and Health Products, of which content is adapted to patients.

## Oversight and monitoring

### Composition of the coordinating center and trial steering committee {5d}

Not applicable

### Composition of the data monitoring committee, its role, and reporting structure {21a}

Due to the characteristics of the study, a phase III study with known drugs (colchicine versus standard treatment), without placebo, an independent data monitoring committee has not been established. Patient safety will be reviewed at quarterly monitoring visits through pharmacovigilance.

### Adverse event reporting and harms {22}

In this study, an adverse event will be defined as any untoward medical occurrence in a subject without regard to the possibility of a causal relationship. The investigators will be responsible for collecting all the adverse events in the clinical history based on those referred by the patient spontaneously or by an interview in the follow-up visits. The causality of the adverse event with the intervention will be evaluated and recorded in the clinical history and in the eCRF.

The investigators will report to the sponsor all serious adverse events occurring to subjects treated in the clinical trial, without undue delay but not later than within 24 h of obtaining knowledge of the events.

The sponsor will report to the Spanish Agency for Medicines and Health Products all relevant information about suspected unexpected serious adverse reactions to investigational medicinal products occurring in this clinical trial.

### Frequency and plans for auditing trial conduct {23}

The CRA is a person independent of the research team who is not involved in patient recruitment and follow-up. The CRA will verify 100% of the events and adverse reactions (pharmacovigilance) that occur in all the follow-up visits and the rest of the variables described in the protocol including adherence to study medication. A monitoring plan is established for the follow-up of the clinical trial. The monitoring plan is designed to include at least one initiation and one close visit, and 4 monitoring visits per year.

### Plans for communicating important protocol amendments to relevant parties (e.g., trial participants, ethical committees) {25}

All notifications defined as relevant or non-relevant amendments will be made to the Spanish Agency for Medicines and Health Products and to the Ethics Committee of reference in accordance with Spanish legislation on clinical trials RD 1090/2015 of December 4, which regulates clinical trials with medicinal products, the Ethics Committees for Research with medicines and the Spanish Registry of Clinical Trials.

The COLCHICOVID study has been obtained the following approvals by the AEMPS and by the EC of Cantabria: initial approval on 06/05/2020 with protocol version 2.2 of 29/04/2020 and the subsequent amendment generating version 3.0 of 22/09/2020.

## Dissemination plans {31a}

The results obtained will be published in journals of impact and in scientific congresses related to the subject of the study.

## Discussion

Colchicine is an old and known drug for the treatment of other rheumatological, cardiovascular, and viral diseases. Due to its anti-inflammatory and anti-fibrotic properties, inhibitory actions on virus replication, and inhibitory effect on coagulation activation, it could be used as an effective treatment for the prevention of coronavirus in patients over 60 years of age.

An early treatment with colchicine for a group of population vulnerable to the infection by COVID-19 could avoid serious complications and reduce the lethality of the disease and also decrease hospital pressure.

Currently, the treatments with relative efficacy used for COVID-19 infection are at the hospital level, but other early treatments that avoid hospital admission have not been described.

The demonstration that colchicine is capable of reducing the complications of SARS-CoV-2 infection will allow an early treatment, easy to prescribe and manage in the primary care setting and at a very low cost.

To this end, we propose a pragmatic clinical trial, adapted to routine practice, to clinically test an early therapeutic strategy in a population highly susceptible to the respiratory complications of SARS-CoV-2 (patients over 60 years). The clinical effectiveness may mitigate the health crisis and prevent the collapse of the health system with the successive waves of the coronavirus pandemic.

## Trial status

The current version of the protocol is 3.0 of the 22nd of September 2020.

The first patient included in the study occurred in August 2020.

The recruitment period is 1 year (August 2021).

## Data Availability

The data obtained in the trial are under the control of the sponsor and the principal investigator of the clinical trial. There are no agreements with other entities.

## Abbreviations

eCRF: Electronic case report form
CRA: Clinical research associate
SCReN: Spanish Clinical Research Network
IDIVAL: Institute of Research Valdecilla
AEMPS: Spanish Agency of Medicines and Medical Devices
EC: Ethics Committee
COVID-19: Coronavirus
ECBNI: Low intervention level clinical trial
